# Supplementary material for: Metallization and molecular dissociation of dense fluid nitrogen
Source: Nat Commun. 2018 Jul 6;9:2624. doi: 10.1038/s41467-018-05011-z (PMC6035179; doi:10.1038/s41467-018-05011-z)
Supplement: Supplementary file 1 — Supplementary Information [file 41467_2018_5011_MOESM1_ESM.pdf]

Supplementary Information for

Metallization and molecular dissociation of dense fluid nitrogen

Shuqing Jiang et al.

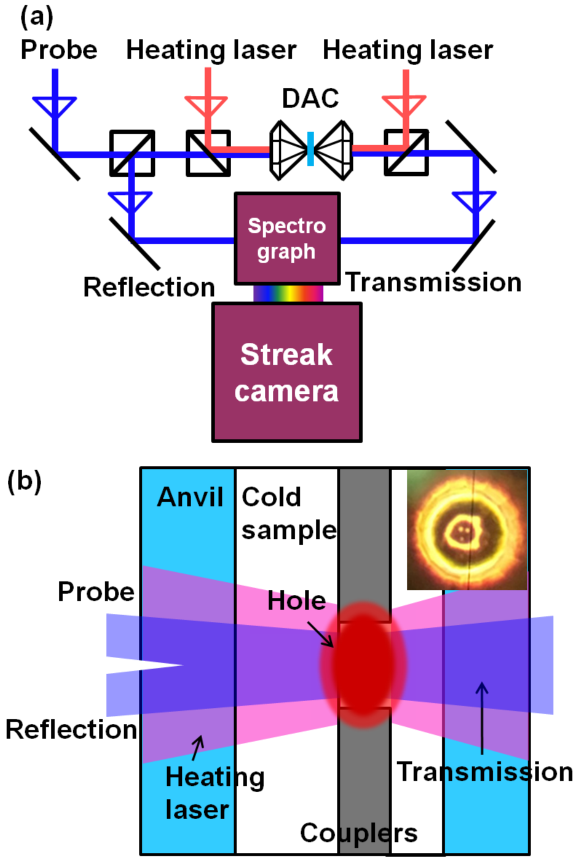

Supplementary Figure 1. Configuration of a single shot laser heating experiment of nitrogen to probe transient transmission and reflection in the diamond anvil cell. (a) Optical schematic: time resolved reflection or transmission can be measured one at a time superimposed with the thermal emission- see our previous publications [1,2] for the timing of acquisition of the optical probes and thermal emission using a streak camera ; (b) Microscopic view of the diamond cell cavity, which contained the nitrogen sample and a metal Ir foil (coupler) which had a small hole(s) (5~10 μm in diameter); coupler is heated by an IR laser from both sides and it converts laser radiation to heat, which is transferred to the adjacent sample inside the hole (if the laser heats the rim of the hole) or from both sides of the coupler (if a flat regular surface is exposed). The transmission probe is passing through the nitrogen sample inside the hole in a coupler, and the reflection probe is detected from the same side as the incident probe beam.

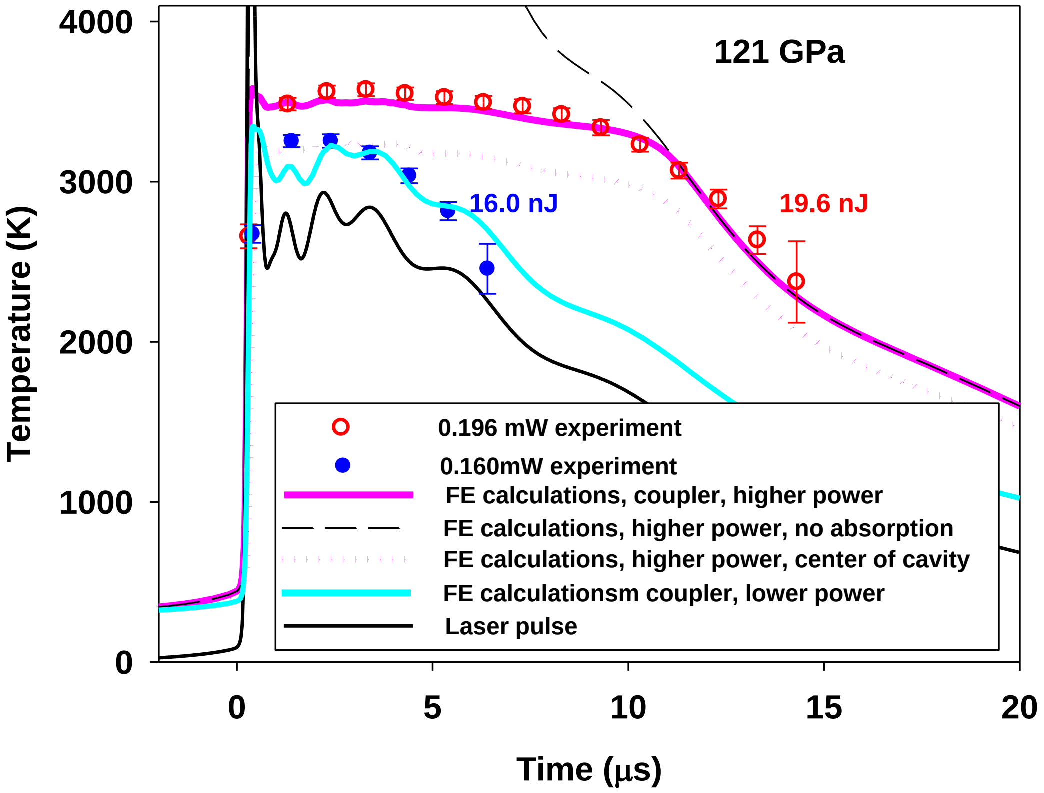

Supplementary Figure 2. Finite element (FE) calculations of the temperature histories in nitrogen at 121 GPa in comparison with the experimentally determined dependencies. Experiments show a plateau, which becomes longer with the increase in heating laser pulse energy (tabulated and measured through the pulsed laser energy). FE calculations picture well this observations. If no absorption is introduced, FE calculations show much higher temperatures instead of plateau approximately corresponding to the onset of the sample absorption. The laser pulse intensity is shown in arbitrary units.

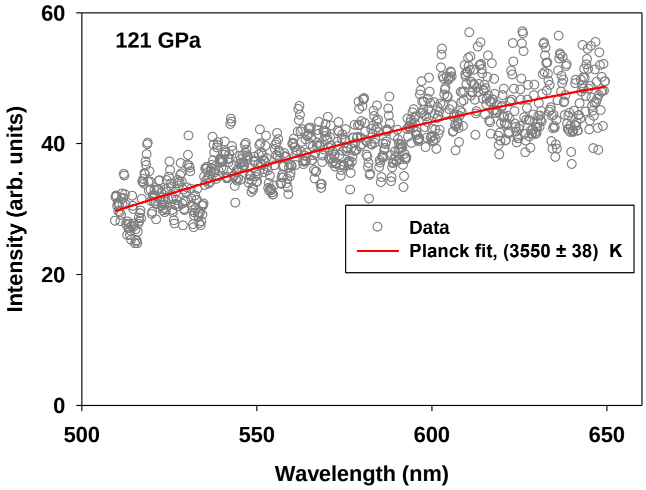

Supplementary Figure 3. The thermal radiation spectrum near the maximum (at the 2 μs, Fig. 1 (a), the bottom panel) used to determine the sample temperature.

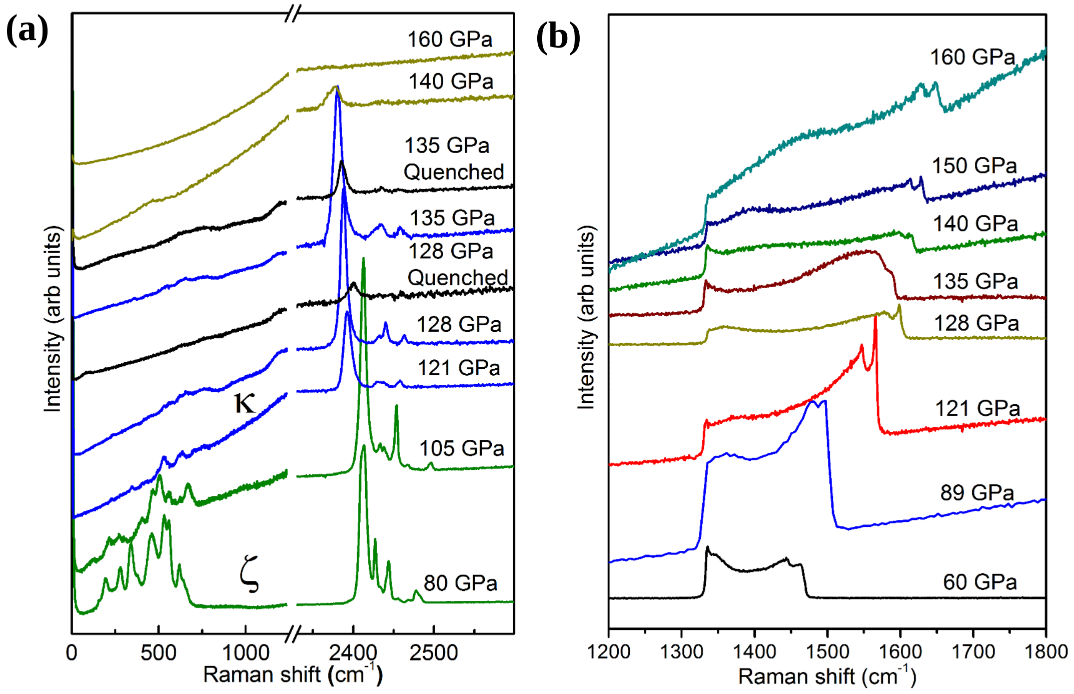

Supplementary Figure 4. The Raman spectra of nitrogen at various pressures before and after laser heating experiments (a) and stressed diamond anvils (b). The pressure was determined using the spectral positions of the N-N stretching mode of N_2 (vibron) [3] (a) and stressed diamond [4] (b) measured by Raman spectroscopy. At above 121 GPa N_2 is in the κ molecular phase [5]. The black lines show the Raman spectra after laser heating at 128 and 135 GPa, which demonstrate a gradual transformation into amorphous η state [6] but no sign of cg or LP nonmolecular nitrogen [7,8].

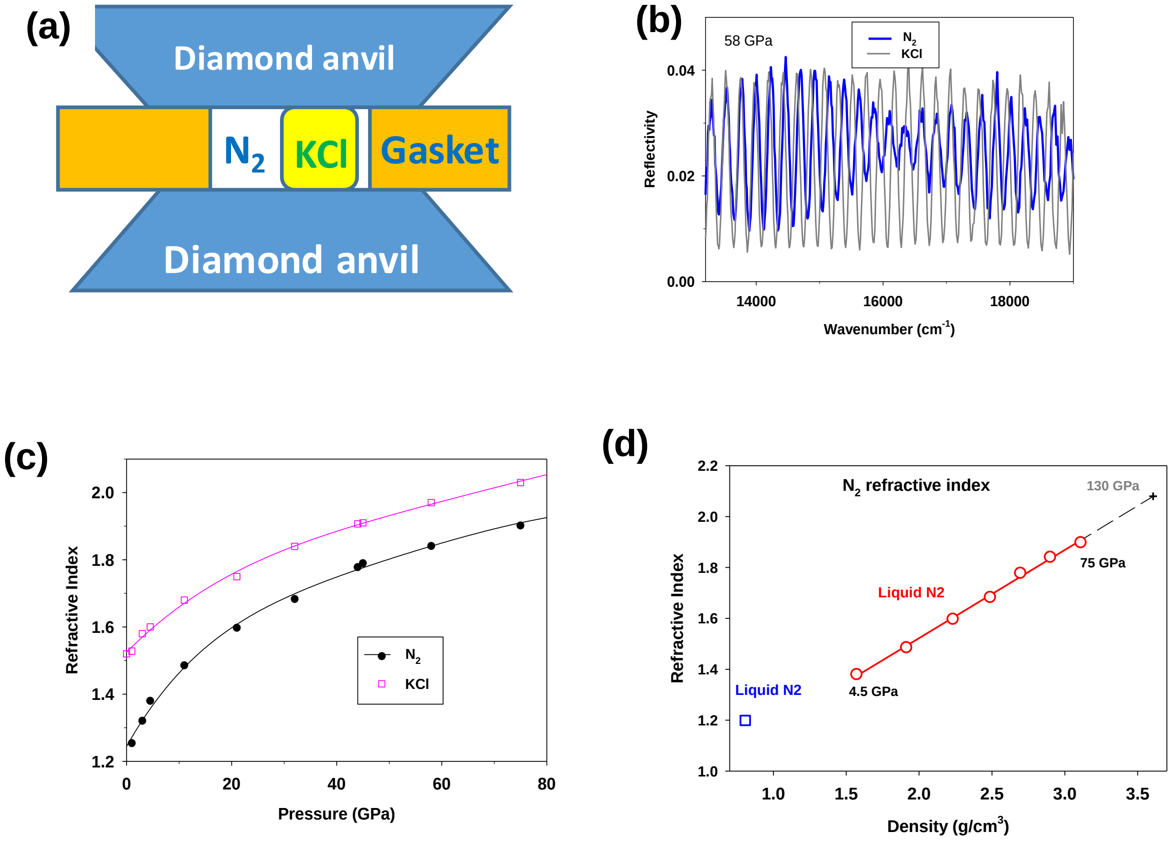

Supplementary Figure 5. Refractive index of nitrogen to 75 GPa (a) Experimental arrangement: a thin slab of KCl is squeezed between diamond anvils and N_2 serves as a pressure medium, reflectivity spectra are measured from the KCl and N_2 regions yielding a series of interference fringes due to interference from the two sample-diamond interfaces, the refractive index of N_2 is determined at each pressure point by referencing to the refractive index of KCl, which is determined by a linear extrapolation as a function of density of measurements of Ref. [9] using the equation of state of Ref. [10] ; (b) Examples of optical interference at high pressures; (c) Results up to 75 GPa as a function of pressure; (d) results extrapolated to 130 GPa using a linear dependence on the density; the equation of state of Ref. [5] is used.

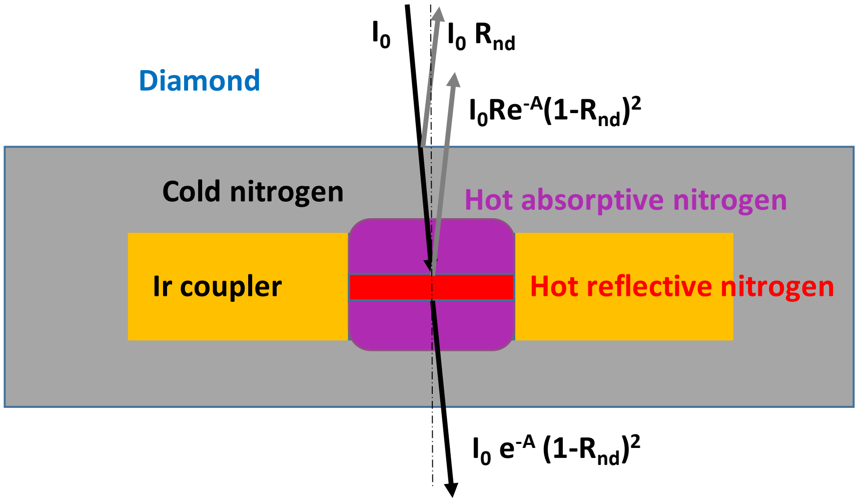

Supplementary Figure 6. A schematic of absorption and reflectivity measurements of liquid conducting nitrogen in the DAC. Hot conducting nitrogen is created in a hole of a metallic Ir coupler. The transmission spectra determine the absorbance *A*. Once nitrogen transforms to a strongly reflective state in the hottest area of the cell, a phase boundary appears between the metallic and semiconducting (absorptive) states. To extract the reflectance spectra we normalize for the optical absorption in semiconducting nitrogen.

Supplementary references

1 McWilliams, R. S., Dalton, D. A., Konôpková, Z., Mahmood, M. F. & Goncharov, A. F. Opacity and conductivity measurements in noble gases at conditions of planetary and stellar interiors. *Proceedings of the National Academy of Sciences of the United States of America* **112**, 7925-7930 (2015).

2 McWilliams, R. S., Dalton, D. A., Mahmood, M. F. & Goncharov, A. F. Optical Properties of Fluid Hydrogen at the Transition to a Conducting State. *Phys Rev Lett* **116**, 255501 (2016).

3 Goncharov, A. F., Gregoryanz, E., Mao, H.-k., Liu, Z. & Hemley, R. J. Optical Evidence for a Nonmolecular Phase of Nitrogen above 150 GPa. *Phys Rev Lett* **85**, 1262-1265 (2000).

4 Akahama, Y. & Kawamura, H. Pressure calibration of diamond anvil Raman gauge to 310GPa. *Journal of Applied Physics* **100**, 043516 (2006).

5 Gregoryanz, E. *et al.* High P-T transformations of nitrogen to 170GPa. *The Journal of Chemical Physics* **126**, 184505, (2007).

6 Gregoryanz, E., Goncharov, A. F., Hemley, R. J. & Mao, H.-k. High-pressure amorphous nitrogen. *Phys Rev B* **64**, 052103 (2001).

7 Eremets, M. I., Gavriliuk, A. G., Trojan, I. A., Dzivenko, D. A. & Boehler, R. Single-bonded cubic form of nitrogen. *Nat Mater* **3**, 558-563 (2004).

8 Tomasino, D., Kim, M., Smith, J. & Yoo, C.-S. Pressure-Induced Symmetry-Lowering Transition in Dense Nitrogen to Layered Polymeric Nitrogen (LP-N) with Colossal Raman Intensity. *Phys Rev Lett* **113**, 205502 (2014).

9 Johannsen, P. G. *et al.* Refractive index of the alkali halides. II. Effect of pressure on the refractive index of 11 alkali halides. *Phys Rev B* **55**, 6865-6870 (1997).

10 Dewaele, A. *et al.* High-pressure -- high-temperature equation of state of KCl and KBr. *Phys Rev B* **85**, 214105 (2012).
